# An Effective Community–Academic Partnership to Extend the Reach of Screenings for Fall Risk 

**DOI:** 10.5888/pcd10.120213

**Published:** 2013-08-22

**Authors:** Lori A. Schrodt, Kathie C. Garbe, Rebecca Chaplin, Jan Busby-Whitehead, Tiffany E. Shubert

**Affiliations:** Author Affiliations: Kathie C. Garbe, University of North Carolina at Asheville, Asheville, North Carolina; Rebecca Chaplin, Land-of-Sky Regional Council, Asheville, North Carolina; Jan Busby-Whitehead, Tiffany E. Shubert, Center for Aging and Health, University of North Carolina at Chapel Hill, Chapel Hill, North Carolina.

## Abstract

Older adults should be screened for fall risk annually. Community providers (people without formal medical training who work with older adults in senior centers or aging services) may be a viable group to expand the reach of screenings. Our community–academic partnership developed a program to increase and assess fall risk screenings by community providers. Community sites hosted training workshops and screening events. Community screenings were well attended and received by providers and older adults. With administrative support from the regional fall prevention coalition and technical support from academia, community providers screened 161 older adults from a broad geographic area. Twenty-one community providers completed the training. Knowledge and confidence surveys demonstrated improvements before and after training (*P* < .001). Skills assessments demonstrated mastery of most skills, but some providers required additional training. Provider feedback indicated screening procedures were complex. Future projects will examine this model using simplified screening procedures.

## Introduction

A fall is a sentinel event for an older adult (1,2). Early identification of older adults at risk can decrease risk of a fall or a fall-related injury (3,4). Each fall prevented reduces health care expenditures and can improve quality of life (5).

Clinical guidelines recommend annual fall risk screening for adults aged 65 or older (6,7). Evidence-based screening tools are available to health care providers (6,7). However, training only health care providers may not meet the demand of the growing older adult population, especially in rural areas or areas where there is a shortage of health care providers (8). These areas often rely on lay “community providers” to perform preventive screenings. Community providers (common job titles include aging program specialist/coordinator, recreation program or wellness coordinator, senior center director/coordinator) typically work in senior or community centers, congregate meal sites, or senior housing. Their responsibilities include providing education and organizing exercise, social, nutrition, and wellness programs. Community providers typically do not have health care backgrounds, but given their work setting, they may have the opportunity to play a major role in fall prevention.

The growing older adult population (9), the financial burden of falls, the effect on quality of life (10), and the scarcity of resources (11) demand creation and broad implementation of innovative systems to prevent falls. Building on existing infrastructures to disseminate fall risk screening is an efficient option to shift focus from postfall medical interventions to prevention strategies.

No models for widespread community-based fall risk screening exist. Typical screenings are small-scale, offered as isolated events at a single site (commonly 1 to 2 times a year) hosted by health care providers (usually physical or occupational therapists). Sporadic events have limited reach; a single provider during a few hours of donated time can only provide services to a small number of people. The isolated event model commonly lacks the infrastructure needed for community outreach and a standardized screening process and communication with primary care providers, which are necessary to create an effective fall risk management continuum.

Community providers may be strategically positioned to implement fall risk screening. Increasing access to screenings may facilitate screening of larger numbers of older adults. Community providers have the advantage of routinely interacting with older adults on a daily or weekly basis during the course of other programming (eg, daily meals, weekly social or exercise programs). The familiarity and comfort older adults often have with community providers may increase screening event attendance and adoption of recommendations.

## Community Context

Western North Carolina is composed of 16 counties along the Appalachian Mountain Range. Fifteen of these counties include an area or population designated by the Health Research and Services Administration as primary care shortage areas, including 2 with designations for the entire county (12). Ten counties have 20% of the population older than age 65 (13), and 10 counties are identified as “hot spots” with fall-related mortality ranging from 9.24 to 17.96 per 100,000 population, compared with the overall North Carolina rate of 8.10 (14,15). The combination of demographics and falls rates has resulted in this region leading the state in fall-related deaths (14,15).

In 2009, the Area Agency on Aging, health care institutions, community service providers, academic institutions, and concerned citizens formed the Western North Carolina Fall Prevention Coalition (Coalition) to reduce the number of falls and fall-related injuries. The Coalition conducted a needs assessment via its 29 partner agencies in early 2010 and determined a need to identify older adults at increased risk for falling and direct them to appropriate prevention services. To meet this need, the Coalition proposed a project to sponsor and organize community screenings and determine whether community providers could accurately and safely perform an evidence-based fall risk screen at community sites. This project was to be conducted in conjunction with National Falls Prevention Awareness Week programming during September 2010.

The objectives of this pilot study were to determine whether 1) there was interest among community organizations to provide a venue and support staff to host fall risk screenings conducted by community providers, 2) there was interest among older adults to participate in fall risk screenings at community sites, and 3) trained community providers could safely and proficiently implement an evidence-based fall risk screening algorithm.

## Community Organization Interest to Host Screenings

The Coalition recruited community sites to host training workshops and screening events, recruited community providers to be trained and conduct screenings, provided organizational and logistical support for training workshops and screening events, developed screening event procedures and forms, developed community-specific resource guides for fall prevention services, and conducted site follow-up. The researchers developed and delivered training workshops, assessed community providers’ performance at the workshop and onsite at screening events, and evaluated outcomes. The Western Carolina University Institutional Review Board approved this study.

The Coalition and the Area Agency on Aging recruited sites and community providers through existing networks, (eg, the Coalition’s website, newsletters, meeting announcements) and via telephone calls and e-mail directly to congregate meal site supervisors and senior centers during June and July 2010. The Coalition’s goal was to recruit 10 sites and 20 community providers to screen 150 older adults. Sites provided space, tables, and chairs for screening events, sent 1 to 2 staff to a training workshop, and promoted the event locally. The Coalition recruited additional community providers by word of mouth. Community providers were required to attend a 3-hour training workshop, conduct screenings at a minimum of 1 community event, and participate in posttraining and onsite evaluations.

We assessed community organization interest for providing a venue and staff to host screening events via uptake by sites and providers and through formal and informal feedback. An Internet link to a follow-up survey was sent by e-mail to all community providers for training and screening event feedback in October 2010. Informal feedback was obtained during screening events and Coalition meetings.

## Older Adult Participation in Screenings

The Coalition advertised screening events through free news media and National Falls Prevention Awareness Week flyers disseminated via Coalition members. Each site received flyers and was asked to recruit older adults.

Attendance at each screening was recorded. Older adults screened were asked to complete an onsite survey assessing overall satisfaction with screening events, whether they would recommend the screening to friends, and whether they would attempt to decrease their fall risk as a result of attending the event.

## Implementation of Screening Algorithm by Community Providers

We offered 2 separate 3-hour training workshops, 2 and 3 weeks (August and September 2010) before screening events. Community providers were required to attend 1 workshop.

Researchers introduced the purpose of the training, reviewed the associated research project, and stressed that research participation was voluntary before beginning the training. All community providers agreed to volunteer for the study and signed an approved informed consent form. Participants completed demographic information and pretraining and posttraining written knowledge and confidence assessments during the workshop.

Written knowledge and confidence assessments and checks of performance-based screening skills assessed whether trained community providers could safely and proficiently implement an evidence-based fall risk screening algorithm. The pretraining and posttraining written knowledge test included 16 multiple choice and true/false questions about risk factors for falls and screening procedures. Each correct response was awarded 1 point, with a maximum score of 16 possible. The pretraining and posttraining screening confidence survey posed 2 case studies. Participants rated their confidence on a scale of 0 to 10 (0 = no confidence, 10 = extremely confident) to 1) ask the correct questions and 2) use a physical performance test to conduct a fall risk screening. We used the average confidence rating across case studies for analysis.

Researchers led the training workshop (n = 3). Content included risk factors for falls, demonstration of the screening algorithm, and hands-on practice of screening skills. We implemented a fall risk screening algorithm developed by the North Carolina Falls Prevention Coalition (NCFPC) and based on the American Geriatrics Society 2001 screening guidelines (6). The algorithm included standardized screening questions and simple timed measures of mobility (Timed Up and Go) (16,17) and balance (single leg stance) (18). Timed objective measures reduced the need for community providers to subjectively evaluate task performance, which is a skill better suited for health care providers (Figure).

**Figure:**
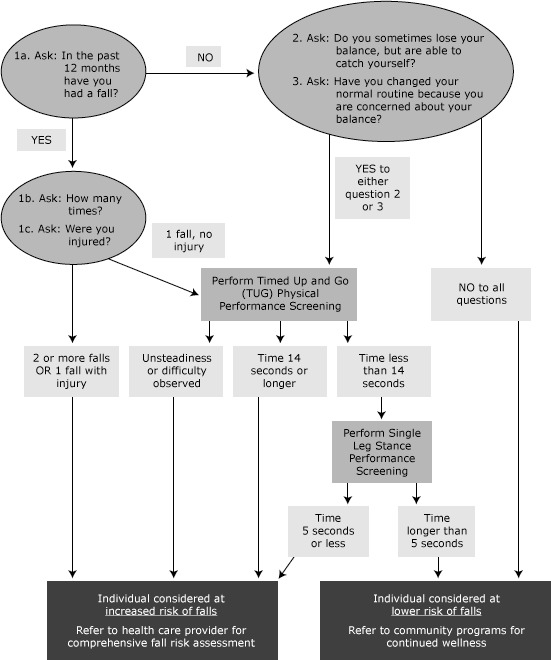
Original 2010 North Carolina Falls Prevention Coalition (NCFPC) Fall Risk Screening Algorithm (later revised). Provided by the NCFPC for use by trained health care and community service providers.

**Table:** 

The figure outlines the flowchart process for fall risk screening. The process begins with simple questions about fall history and balance. Depending on responses to the questions, individuals will either be directly identified as having an increased or lower risk for falls, or be directed to undergo physical performance screening. Scores on physical performance screening tests are then used to identify individuals as at an increased or lower risk of falls. Individuals identified at an increased risk of falls are referred to their health care provider for a comprehensive fall risk assessment, while those identified as at a lower risk of falls are referred to community programs for continued wellness.
The algorithm starts with question 1a, which is to ask the person being screened, “In the past 12 months have you had a fall?” Response options are yes or no. If no, proceed to questions 2 and 3. If yes, proceed to questions 1b and 1c.
Question 1b is to ask the individual, “How many times?” and Question 1c is to ask the individual, “Were you injured?” If responses to questions 1b and 1c indicate 1 fall but no injury, then perform the Timed Up and Go (TUG) Physical Performance Screening. Proceed to TUG outcomes below.
If responses to questions 1b and 1c indicate 2 or more falls or 1 fall with injury, the person is considered at an increased risk of falls. TUG is not performed and the person is referred to their health care provider for comprehensive fall risk assessment.
Question 2 is to ask the person being screened, “Do you sometimes lose your balance, but are able to catch yourself?” Question 3 is to ask, “Have you changed your normal routine because you are concerned about your balance?” If responses to questions 2 and 3 are both no, then the person is considered at lower risk of falls. TUG is not performed and the person is referred to community programs for continued wellness. If the response to either question 2 or 3 is yes, then perform the TUG.
In the TUG screening, if the person being screened is unsteady or has difficulty performing the test or if time to complete the TUG is 14 seconds or longer, then the person is considered at increased risk of falls. Refer him or her to a health care provider for comprehensive fall risk assessment. If time to complete the TUG is less than 14 seconds, then the person is instructed to perform the Single Leg Stance Performance Screening.
In the Single Leg Stance Performance Screening, if time for single leg stance is 5 seconds or less, then the person is considered at increased risk of falls. Refer him or her to a health care provider for comprehensive fall risk assessment. If time for single leg stance is longer than 5 seconds, the person is considered at lower risk of falls. Refer him or her to community programs for continued wellness.

Each participant received handouts detailing procedures for conducting screenings, organizing and conducting screening events, and completing necessary forms. Training workshops included discussion of event logistics such as organization (eg, number and type of chairs and tables, traffic flow, documents needed), marketing, and safety. Orientation to county-specific fall prevention resource guides was provided. For this pilot program, participants performed all aspects of the event, including check-in (older adult registration and consent), fall risk screening, and check-out (standardized letters to each older adult’s physician and resources based on fall risk category). Participants received equipment necessary to perform screenings at community events, including equipment for the Timed Up and Go (16,17) (a 10-foot piece of rope for measuring walking distance, tape to mark walking distance on the floor, and a stopwatch) at the workshop conclusion.

Participants practiced hands-on screening algorithm administration by using case studies and were provided with feedback by researchers during training. Once each participant felt confident with the procedures, they completed a skills assessment by screening a simulated case role-played by the researchers. Researchers assessed screening proficiency via a standardized skills checklist. Participants’ ability to implement the algorithm was scored according to the following criteria: 1) asking questions in the correct sequence, 2) conducting performance tests when indicated (including proper and safe administration), 3) conducting performance tests in correct sequence, and 4) identifying the correct risk category and providing appropriate recommendations (follow-up with physician or community programs) based on risk category. One point was awarded for fulfillment of each criterion. We converted scores to percentage correct on the basis of points achieved out of a maximum of 4 points. Participants who made errors received feedback and were encouraged to repeat the skills assessment with another case study.

Researchers conducted a final skills assessment onsite during screening events (September 2010). One researcher attended each event and assessed participants’ ability to implement the screening algorithm and protocols and provided additional onsite training as necessary. To assess retention, the standardized skills checklist was conducted with a convenience subsample of 13 participants during their first onsite screening.

Researchers conducted statistical analyses to examine participants’ knowledge, confidence, and screening skill proficiency. Descriptive statistics examined participant characteristics and results of posttraining and onsite screening event skills assessments. Paired *t* tests examined differences in the results of the written knowledge test and confidence rating before and after the training by using IBM SPSS Statistics for Windows, version 19.0 (IBM Corp, Armonk, New York). A *P *value of less than .05 determined significance.

## Outcomes

Community support and interest indicated that organizations and older adults were interested in fall risk screenings at community sites. Twelve sites (senior and community centers, congregate meal sites, senior housing, and a hospital) in 5 counties agreed to host a fall risk screening event and committed staff to the program. Community organization uptake exceeded the Coalition’s goals for this pilot. All sites identified by the Coalition agreed to participate. Eight additional organizations, including 1 with sites in 2 counties not served during this 2010 project, expressed interest in training staff and hosting future fall risk screenings. Follow-up survey comments from community providers indicated that providers viewed events as a positive contribution to their communities and they liked having a new skill set to offer clients.

The 12 sites screened 161 older adults, also exceeding the Coalition’s goal. Of older adults who completed onsite surveys (n = 85), 87% indicated they “strongly agreed” with statements indicating satisfaction with the screening events, facilities, and screeners. Older adults also indicated that they would recommend the screening event to friends (86%) and would make changes to decrease their fall risk as a result of attending the events (75%).

Twenty-nine people attended training to become community screeners. Eight were health care professionals and, thus, excluded from these analyses. Our sample of 21 community providers (mean age, 47; range, 20–76 years) included people from organizations offering community education, aging services, nutrition and dining programs, and fitness services. Three volunteers were local university students (Table 1).

**Table 1:** Characteristics of Community Providers Who Attended Training for Fall Risk Screenings (N = 21), North Carolina, 2010

Characteristic	No. of Participants^a^
**Sex**	
Female	18
Male	3
**Age ≥65 y**	6
**Highest education level completed**	
High school	4
Associate degree	2
Bachelor degree	9
Graduate degree	4
**Prior experience**	
Screening for fall risk	2
Educating older adults	17
Organizing programs for older adults	18
Attended other fall prevention education	10
**Self-reported history of falls**	2

a Numbers may not equal total because of missing data.

Participants significantly improved in their written knowledge (*t*_20_ = −7.56, *P* < .001) and screening confidence immediately posttraining (*t*_20_ = −8.94, *P* < .001). Posttraining skills assessments demonstrated that 14 participants scored 100% on the skills assessment criteria, 6 scored 75%, and 1 scored 50%. Skills assessments conducted during onsite events indicated 10 of the 13 participants scored 100% on the assessment criteria, 2 scored 75%, and 1 scored 50% (Table 2). Participant feedback, via formal survey and informal conversation, supported the researchers’ assessment that the algorithm was too complex and that following the screening sequence posed a challenge for these community providers.

**Table 2:** Results of Community Provider Posttraining Skills Assessment (N = 21) and Onsite Screening Event Skills Assessment (N = 13) for Fall Risk Screening, North Carolina, 2010

Screening Skills Assessment Criteria	No. Demonstrating Mastery		No. Needing Additional Training	
	Posttraining	Onsite Event	Posttraining	Onsite Event
Asks questions in correct sequence	19	12	2	1
Conducts performance tests when indicated (includes proper and safe administration)	17	12	4	1
Conducts performance tests in correct sequence	21	13	0	0
Identifies correct risk category and provides appropriate recommendations	19	11	2	2

## Lessons Learned and Implications for Future Dissemination

Results from this pilot suggest minor training modifications and simplification of the algorithm would better ensure future success. Several lessons learned emerged from follow-up surveys and informal feedback. First, the required equipment (chairs) to host screening events sometimes posed a challenge. The Timed Up and Go (16,17) requires a standard height chair with armrests. Many sites did not have proper chairs readily available. Using a chair without armrests can be unsafe and is inconsistent with protocols for evidence-based time criteria for fall risk. In the future, sites will be given guidance and resources to ensure all equipment is onsite before screening events. Second, participants needed more practice time during training to become proficient at administering the screening algorithm. Future training workshops will allocate more time for hands-on practice. Third, community providers should screen negative for fall risk. Researchers did not anticipate the need to require community providers to meet a minimum level of physical ability. The next iteration of screener requirements will clarify the physical skills required to better ensure safety. Fourth, the algorithm was challenging for community providers to follow accurately and consistently. The algorithm aimed to capture mobility and balance impairments in a wide range of older adults and exempt certain people from mobility and balance screening either for safety reasons (high fall risk) or for time (too high functioning, low fall risk). However, some community providers needed assistance to conduct screenings, indicating that the algorithm was too complex. Feedback from older adults indicated that they wanted to undergo the mobility and balance screening even if they exempted out of testing. The Coalition has now worked with the NCFPC to simplify the algorithm. Fifth, participants reported that the time lag between training and screening events was too long. They recommended scheduling training a week before screening events. Participants also recommended each host site provide a volunteer to assist with paperwork to improve time management and flow. These logistic changes will be addressed in future scheduling and staffing.

The objectives of this community engagement effort were met. Fall risk screening events were well received and empowering for the community. Participants enjoyed administering screenings and valued the potential positive impact on their community. This activity provided the community with insight into the issue of falls and increased the interest of community providers and older adults regarding fall prevention. Future projects will assess community providers’ ability to use a simplified screening algorithm.

## References

[1] Donald IP , Bulpitt CJ . The prognosis of falls in elderly people living at home. Age Ageing 1999;28(2):121–5. 10.1093/ageing/28.2.121 10350407

[2] Hornbrook MC , Stevens VJ , Wingfield DJ , Hollis JF , Greenlick MR , Ory MG . Preventing falls among community-dwelling older persons: results from a randomized trial. Gerontologist 1994;34(1):16–23. 10.1093/geront/34.1.16 8150304

[3] Gillespie LD , Robertson MC , Gillespie WJ , Lamb SE , Gates S , Cumming RG , Interventions for preventing falls in older people living in the community. Cochrane Database Syst Rev 2009;(2):CD007146. 19370674 10.1002/14651858.CD007146.pub2

[4] Gillespie ND , McMurdo ME . Falls in old age: inevitable or preventable? Scott Med J 1998;43(4):101–3. 9757495 10.1177/003693309804300402

[5] Stevens JA , Corso PS , Finkelstein EA , Miller TR . The costs of fatal and non-fatal falls among older adults. Inj Prev 2006;12(5):290–5. 10.1136/ip.2005.011015 17018668 PMC2563445

[6] American Geriatrics Society, British Geriatrics Society, and American Academy of Orthopaedic Surgeons Panel on Falls Prevention. Guideline for the prevention of falls in older persons. J Am Geriatr Soc 2001;49(5):664–72. 10.1046/j.1532-5415.2001.49115.x 11380764

[7] American Geriatrics Society/British Geriatrics Society Clinical Practice Guideline: prevention of falls in older persons. American Geriatrics Society; 2010. http://www.americangeriatrics.org/health_care_professionals/clinical_practice/clinical_guidelines_recommendations/2010/. Accessed June 30, 2012.

[8] Shortage designation: health professional shortage areas and medically underserved areas/populations. Health Resources and Services Administration; 2012. http://www.hrsa.gov/shortage/. Accessed July 10, 2012.

[9] Howden LM , Meyer JA . Age and sex composition: 2010 census briefs. United States Census Bureau; 2011. http://www.census.gov/population/age/. Accessed June 14, 2013.

[10] Stevens J . Cost of falls among older adults; 2011. http://www.cdc.gov/HomeandRecreationalSafety/Falls/fallcost.html. Accessed June 30, 2012.

[11] Retooling for an aging America: building the health care workforce. Washington (DC): National Academies Press; 2008. 25009893

[12] Primary medical care health professional shortage areas (HPSAs) designated as of April 1, 2012. Health Resources and Services Administration; 2012. http://bhpr.hrsa.gov/shortage/updateddesignations/index.html. Accessed June 30, 2012.

[13] A profile of people age 60 and over in North Carolina. Raleigh (NC): Division of Aging and Adult Services; 2011.

[14] Stevens C . Older adult injuries in North Carolina 2004–2007. Raleigh (NC): Department of Health and Human Services; 2010.

[15] North Carolina Institute of Medicine. Healthy North Carolina 2020: a better state of health. Morrisville (NC): North Carolina Institute of Medicine; 2011.

[16] Shumway-Cook A , Brauer S , Woollacott M . Predicting the probability for falls in community-dwelling older adults using the Timed Up and Go Test. Phys Ther 2000;80(9):896–903. 10960937

[17] Podsiadlo D , Richardson S . The timed “Up & Go”: a test of basic functional mobility for frail elderly persons. J Am Geriatr Soc 1991;39(2):142–8. 1991946 10.1111/j.1532-5415.1991.tb01616.x

[18] Vellas BJ , Wayne SJ , Romero L , Baumgartner RN , Rubenstein LZ , Garry PJ . One-leg balance is an important predictor of injurious falls in older persons. J Am Geriatr Soc 1997;45(6):735–8. 9180669 10.1111/j.1532-5415.1997.tb01479.x

